# Mission Planning and Decision Support for Underwater Glider Networks: A Sampling on-Demand Approach

**DOI:** 10.3390/s16010028

**Published:** 2015-12-26

**Authors:** Gabriele Ferri, Marco Cococcioni, Alberto Alvarez

**Affiliations:** 1 Research Department, NATO STO Centre for Maritime Research and Experimentation (CMRE), Viale San Bartolomeo 400, 19126 La Spezia, Italy; Alberto.Alvarez@cmre.nato.int; 2 Dipartimento di Ingegneria dell’Informazione, University of Pisa, Largo Lucio Lazzarino 1, 56122 Pisa, Italy; marco.cococcioni@iet.unipi.it

**Keywords:** glider networks, sensor networks, sampling on-demand, optimal sampling, MyOcean forecasts provider, data assimilation

## Abstract

This paper describes an optimal sampling approach to support glider fleet operators and marine scientists during the complex task of planning the missions of fleets of underwater gliders. Optimal sampling, which has gained considerable attention in the last decade, consists in planning the paths of gliders to minimize a specific criterion pertinent to the phenomenon under investigation. Different criteria (e.g., *A*, *G*, or *E* optimality), used in geosciences to obtain an optimum design, lead to different sampling strategies. In particular, the *A* criterion produces paths for the gliders that minimize the overall level of uncertainty over the area of interest. However, there are commonly operative situations in which the marine scientists may prefer not to minimize the overall uncertainty of a certain area, but instead they may be interested in achieving an *acceptable* uncertainty sufficient for the scientific or operational needs of the mission. We propose and discuss here an approach named *sampling on-demand* that explicitly addresses this need. In our approach the user provides an objective map, setting both the amount and the geographic distribution of the uncertainty to be achieved after assimilating the information gathered by the fleet. A novel optimality criterion, called $A_{\eta }$, is proposed and the resulting minimization problem is solved by using a Simulated Annealing based optimizer that takes into account the constraints imposed by the glider navigation features, the desired geometry of the paths and the problems of reachability caused by ocean currents. This planning strategy has been implemented in a Matlab toolbox called SoDDS (Sampling on-Demand and Decision Support). The tool is able to automatically download the ocean fields data from MyOcean repository and also provides graphical user interfaces to ease the input process of mission parameters and targets. The results obtained by running SoDDS on three different scenarios are provided and show that SoDDS, which is currently used at NATO STO Centre for Maritime Research and Experimentation (CMRE), can represent a step forward towards a systematic mission planning of glider fleets, dramatically reducing the efforts of glider operators.

## 1. Introduction

The capability to understand, predict and reconstruct accurately the ocean dynamics is crucial for many oceanic applications, such as eco-system based fisheries, pollution management and effective planning and execution of naval/underwater operations.

Oceans, however, are extremely complex dynamical systems. Turbulence, typical of marine environments, involves large interactions between a wide range of spatiotemporal scales [1]. Physical spatiotemporal variability deeply affects oceanic chemical and biological processes, [2,3]. Furthermore, the importance of the interactions between the physical, chemical and biological fields increases the difficulty in the study of the marine environment.

These factors define harsh requirements for measurement strategies in order to acquire meaningful information from the data collected at sea:

- Simultaneous measurements of the different physical, chemical and biological parameters are required.
- The measurements need to be taken with adequate spatiotemporal resolution according to the phenomena under investigation.

These requirements make the task of planning and executing sampling campaigns difficult and expensive.

Recent advancements in the field of sensing technologies, micro-electronics and micro-fabrication have enabled the miniaturization of sensors and devices. These results, together with the maturing of robotics and advanced ICT-based technologies, made it possible in the last two decades for the development of new systems for sampling the oceans with an increased spatiotemporal measurement resolution and at lower costs with respect to traditional methods (traditionally, field measurements have been carried out by using dedicated vessels with expert personnel onboard, fixed monitoring stations, e.g. buoys, and, more recently, by remote sensing from space). These new systems (drifters [4], autonomous surface vehicles (ASVs) [5], autonomous underwater vehicles (AUVs) [6] and gliders [7]) are characterized by different range and mobility features and are more and more employed by oceanographers to complement traditional methods. Their features offer new possibilities in collecting measurements in a sustained way and over large geographical areas. This new way of acquiring measurements opens new horizons in sensing and modeling oceans. Trends in research suggest that future ocean observations systems will be constituted of heterogeneous, small, intelligent and cheap platforms [8] constituting networks able to provide sustained synoptic observations (*i.e.*, ability to map ocean structures at adequate spatial resolution faster than significant changes occur).

AUVs propelled by traditional thrusters can cover limited areas due to their intrinsic endurance limits and are well suited to provide hints on local phenomena such as fronts, algae blooms or hydrothermal vent activity [9,10]. Gliders, on the other hand, are characterized by an extended endurance and offer the possibility of long-time sampling mission (in the order of months) in open ocean waters [7]. Gliders do not have propellers for their propulsion, but use buoyancy changes, their hydrodynamic shape, fins and controllable movement of the battery pack to perform vertical zig-zag motions between the surface and a predetermined depth with a net horizontal displacement. The gliders emerge at some locations and communicate via satellite link with a control station: in this way the pilot or software can command the vehicle the next waypoint to be reached. The nominal horizontal speed is approximately 2 km·h${}^{-1}$. Coastal versions of gliders are limited to operate between 10 and 200 m depth while deep ocean versions can reach 1000 m depth. However, the capability of long range missions is paid with a low achievable maximum speed: this may be a big issue for the glider navigation and their navigation planning since water currents can modify their path or may also avoid the possibility to reach some commanded waypoint. This needs to be carefully considered in the generation of the glider path to guarantee the reachability of the planned waypoints.

Theoretical and field research has been conducted in recent years on glider mission planning taking into account their peculiar features [11,12]. *Multi-glider* approaches are particularly effective in sampling missions to increase the coverage of the mission area and to acquire a synoptic view of the oceanographic field of interest. The problem of effectively coordinating the movement of a fleet of gliders arises. This was investigated in [13,14] with the main idea of keeping a given fleet formation during coordinated movements of the group of vehicles.

The key challenge to fully exploit glider capabilities is therefore how to plan the vehicle trajectories to maximize the information content collected by means of the available measurements. The measurements, in fact, are limited in number if compared to the geographical extent of the area of study.

To approach this challenge, *adaptive sampling* strategies have been proposed. In adaptive sampling approaches previous measurements influence the evolution of the sampling survey to increase the amount of acquired information concerning a phenomenon under investigation [15]. This kind of data-driven sampling approaches has been investigated and applied in different scenarios: for measuring with gliders the temperature field in the Ligurian sea [16], for reconstructing a lake surface temperature with an ASV and fixed sensors [17] and for hydrothermal vents prospecting along oceanic ridges with an AUV [9,10]. Adaptive sampling aims at increasing the acquired information by driving the sampling process in the most “interesting” areas.

A key factor in supporting and optimizing the planning can be represented by mathematical models of the oceans [18]. Mathematical ocean models can forecast ocean properties such as currents, salinity, temperature or plankton concentrations, or help in reconstructing fields of ocean parameters [18] from data collected at sea. Research in past decades on ocean models has improved the quality of currently adopted models [19,20,21,22]. Nowadays oceanographic models represent much of the relevant physics [23] and the accuracy in their predictions has notably increased. Nevertheless, numerical models cannot represent all the complexity of ocean dynamics and errors in initial and boundary conditions may be present, potentially compromising the prediction quality. Recent advancements in data assimilation and optimal interpolation showed that models can provide better forecasts if measurements taken on field are assimilated into the running model [24].

Combining modeling and sensing is an effective way to provide useful tools and support to science, engineering and industry. In current research trends, sampling and modeling are more and more related and interconnected. The two processes can offer useful feedback to each other:

- From one side, on field measurements can improve the quality of models through data assimilation and/or data-driven parameters adaptation.
- On the other, models, by quantifying the uncertainty related to their predictions, can guide the sampling campaigns.

Measurements, in fact, can be taken at the locations suggested by the models to maximize the information content and reduce the uncertainty over a certain region.

A possible approach to maximize the information content given a limited number of measurements consists in selecting the sampling locations to optimize some cost functions. A cost function, or sampling metric, must be defined and sets the objectives of the survey (e.g., sampling of regions with the highest oceanography variability, or regions where the model uncertainty is higher). This approach, known as *optimal sampling*, can use the forecasts of expected uncertainty over the quantity under investigation provided by the ocean models. Cost function can be designed to plan paths to minimize the *a posteriori* uncertainty produced after the assimilation of the information brought by the planned measurements. The complexity in optimization of the selected cost function determines if the process can be performed in real-time allowing to change dynamically the path of the vehicles as new information becomes available. Otherwise, if a large time is needed to find a solution, an off-line computation needs to be used to plan the paths before the beginning of the mission.

Bretherton and co-workers [25] provided the first structured approach to optimize the sampling performance of a network of oceanographic sensors. Stimulated by the need to design a current meter array, they used objective mapping to determine the current meter array configuration that produced a mapped field with minimum mean square expected error.

The problem of optimizing the sampling performance of a network of drifters was considered in [4]. In that work, the launching positions of 25 drifters are decided with a Genetic Algorithm based optimization to achieve homogeneous coverage of a portion of the Azores region in space and time. Drifters guarantee extremely long range missions and high endurance, but they cannot control their motion so that a high-level planning of their measurement strategy cannot be achieved.

More recently the maneuverability offered by autonomous underwater vehicles (AUVs) can be used for driving the vehicles to sample along optimal paths. In [26] an approach based on Mixed Integer Linear Programming was used to mininize the path integral of the uncertainty values along the path vehicles. In [23] genetic algorithms are used to find paths for the vehicles minimizing a cost function composed of different components such as oceanographic variability along the trajectory, oceanographic temperature range or integrated uncertainty along the paths.

More recent studies have adopted optimal sampling strategies to minimize uncertainties in the predictions produced by ocean models. Cost functions are built on predicted errors when observations by the vehicles are assimilated into the numerical model [20,27].

Alvarez and Mourre [28] investigated an optimal approach with one fixed mooring and one glider for measuring a temperature field in an area in the Ligurian Sea. In the approach the path of the glider was designed to minimize various norms of the posterior covariance matrix after the assimilation of the measurements considering, at the same time, the constraints imposed by the vehicle’s dynamics and the sea currents. Three different criteria were used to minimize posterior covariance matrix: minimizing the trace (*A* optimal), the maximum diagonal value (*G* optimal) and the maximum eigenvalue (*E* optimal). The results based on model data for the scenario under investigation indicate that the most appropriate strategy for environmental characterization using gliders employs the *A* optimal criterion, minimizing the overall uncertainty over the study area.

A limitation of the *A* optimal criterion is that it does not allow us to specify distinct levels of tolerated uncertainties in different areas. Defining the levels of target uncertainties is a quite common need for marine scientists. In many operative situations they may prefer not to minimize the mean uncertainty of a certain area, but they may be interested in achieving distinct *acceptable* levels of uncertainty in different regions, considered sufficient to meet the scientific and/or operational needs of the overall mission. This may be due, for instance, to the limited number of available sensing assets with respect to the size of the area to cover, or to operational requirements setting a certain pre-fixed uncertainty over some regions which is sufficient for the mission objectives. As an example, it may happen that in some peripheral regions the tolerated maximum uncertainty is higher than that required in the core region of the mission.

This adds the need to drive the sampling to reach an objective uncertainty that may vary geographically in the area of interest. We name this kind of approach **sampling on-demand**, since the sampling strategy is driven by the needs of the mission planners to better meet the real requirements of scientific/operational missions.

In this work we propose a framework for the mission planning of a fleet of gliders based on the sampling on-demand paradigm [29]. To formalize the sampling on-demand paradigm we introduce a variant of the *A* criterion that we have named $A_{\eta }$. In $A_{\eta }$ the classical *A* optimal index is modified to take into account a desired map of target (tolerated) uncertainty, provided by the user at the beginning of the mission planning. The resulting optimization problem is solved by using a Simulated Annealing [30] based optimizer that produce the waypoints for the paths of the gliders of the fleet. The algorithm takes into account the constraints typical of the vehicles and the problems of reachability caused by ocean currents. The proposed method is validated on sea currents and temperature data coming from an ocean mathematical model of an area of study covering the Western Mediterranean Sea.

Our approach provides a method to plan glider missions tailored to the needs of scientists. Furthermore, pursuing the idea of creating an operational tool for scientists, we have integrated the proposed sampling on-demand algorithm in a Matlab (^®^MathWorks) toolbox, named Sampling on Demand and Decision Support (SoDDS) [31]. SoDDS is capable of downloading the forecasts of the ocean fields of interest and the ocean currents from the public available MyOcean repository [32]. The tool provides two graphical user interfaces that allow the user-friendly definition of the sampling area and to define the desired target uncertainties. This tool provides an integrated, effective and operational system for glider operators to ease the whole mission planning process and post-mission analysis.

The paper is organized as follows. In the next section we describe the mathematical formalism of the sampling on-demand approach. In Section 3 we detail the optimization algorithm used to produce the paths of gliders. In Section 4 we describe the software architecture of SoDDS Matlab toolbox and the associated graphical user interfaces to be used by the fleet operators. In Section 5 we discuss the results of the sampling on-demand algorithm by using the SoDDS toolbox in three different scenarios in a study area covering the Western Mediterranean Sea. We conclude by discussing the implications of the presented study and future work in Section 6.

## 2. Mathematical Formalism

The problem addressed here is how to plan glider trajectories to provide the best representation of the mesoscale field in a synoptic scale. Oceanographically, the adequacy of a glider sampling strategy can be measured in terms of the dynamical information that can be extracted from the measured field. This is achieved by estimating, from the data acquired at the sampling locations, the best values at grid points of a regular grid.

To proceed with the mathematical description of the problem, we make the assumption that the measurements are synoptic. No time dependence is thus considered in the following analysis and the quantities related to the field to be investigated are considered static. Procedures could be extended to the case of nonsynopticity, however the computational burden and the information needed about the physical processes in a certain area would be increased without adding significant insight to the addressed problem. Two interesting operational cases are considered corroborating our assumption.

One case considers surveys in the Mediterranean Sea covering a timespan of less than 4–5 days. This time period is generally considered the local synoptic time scale [28], so the acquired measurements can be assimilated and used to reconstruct the field of interest.

Another possibility is to carry out a covariance analysis on a larger time-scale (10–20 days). In this case, even if the temperature field will change in the mission timespan, we can assume with a good approximation that the uncertainty of the model is not changing considerably in the considered time length. Since we are interested in a covariance analysis in the proposed $A_{\eta }$ criterion, we can adopt the same approach and consider a static prior covariance and static mean values for the field under investigation. This is the case of interest for our application that will be considered in this work.

Under static conditions, a common procedure in oceanography to estimate the values of a scalar field at unobserved points is the Gauss-Markov smoothing (also known as linear minimum variance estimate or objective analysis [33]). The sampled field is interpreted as a weakly stationary or second-order stationary process [28] defined by known background $\bar{\psi }\left(x\right)$ (mean values of the field at the grid points x) and covariance matrix

(1) $$Cov\left(\psi \left(x\right)\right)=E[\left(\psi \left(x\right)-\bar{\psi }\left(x\right)\right){\left(\psi \left(x\right)-\bar{\psi }\left(x\right)\right)}^{T}]=\Sigma$$

We assume the observations $\psi^{o}$ are in the form of a linear combination of the state ψ(x), that is $\psi^{o}=H\psi \left(x\right)+v$, with v the unobservable observation noise, assumed as zero-mean and Gaussian, and H the observation matrix, that is, a matrix operator that interpolates from the regular grid to measurement locations. The method computes the linear minimum variance estimate $\hat{\psi }\left(x\right)$ for the field ψ(x) (and the relative error covariance $\hat{\Sigma }$) at given unsampled locations of the regular grid from the observations, the locations where they are collected and the a priori knowledge or in situ estimation of the mean/covariance of the sampled field. The analyzed field and covariance error matrix are obtained by

(2) $$\hat{\psi }\left(x\right)=\bar{\psi }\left(x\right)+K\left(\psi^{o}-H\bar{\psi }\left(x\right)\right)$$

(3) $$\hat{\Sigma }=\Sigma -KH\Sigma$$

where $\hat{\psi }\left(x\right)$ and $\bar{\psi }\left(x\right)$ are respectively the state vectors of analyzed and background fields at gridpoint locations; $\psi^{o}$ is the vector of observations; H is the observation matrix, and K is the gain matrix defined as

(4) $$K=\Sigma H^{T}{\left(H\Sigma H^{T}+V\right)}^{-1}$$

The superscript *T* indicates the transpose operation, and V is the observation error covariance matrix. The latter will be assumed diagonal. It is important to note that to compute the posterior covariance $\hat{\Sigma }$ we need to know only the locations of the measurements, while the knowledge of the values of the observations is not necessary. This allows its computation by planning the sampling locations before the mission is executed. In the present work the ergodic hypothesis is assumed, that is, the average of simulated oceanographic processes over a relatively long period of time and the average over the statistical ensemble are considered equivalent.

The posterior covariance matrix $\hat{\Sigma }$ is a measure of the reliability of field estimates when observations are available at certain locations. For this reason the minimization of some norm of this matrix reduces the uncertainty of the model estimates. A measure of the “magnitude” of a matrix is usually described by a scalar function of the matrix. An optimal design can be therefore described as

(5) $$\psi^{\Gamma }=\underset{\psi^{o}}{\arg\min}\;\;\Gamma \left(\hat{\Sigma }\right),\;\;\;\;\psi^{o}\in \Omega$$

where Γ(•) is a scalar function of the covariance matrix and Ω represents the set of possible measurements given the operational constraints (number of gliders, possible path due to glider navigation features or the influence of sea currents). It remains to define:

- the numerical discretization of the problem into forms that are solvable using linear algebra methods (implying how to build the H matrix);
- a suitable metric of the posterior covariance to be minimized (the Γ(•) function);
- an algorithm that optimizes this metric by selecting the fleet observation locations. In this process, glider operational constraints must be respected along with geometric constraints on the generated paths to produce “smooth” paths.

### 2.1. Discretization Procedure of the Problem

For our scenario, a two-dimensional finite-element mesh regular grid is adopted with the physical domain subdivided into a finite number of quadrilateral area elements. The intersection of two elements is an edge, a corner, or empty. A corner of an element cannot lie on the edge of an adjacent element. The corners of the elements are called the nodes.

A continuous scalar function ψ(x) is then approximated in terms of the nodal values and some interpolation functions within each element,

(6) $$\psi \left(x\right)=\sum_{k=1}^{4}N_{k}\left(r,s\right)\psi_{k}$$

where $\psi_{k}$ is the value of the function at node or grid point *k*, and $N_{k}\left(r,s\right)$ represents the interpolation functions expressed in a local coordinate system {r,s} [34]. Piecewise polynomials of low-order or spline functions are usually employed as the interpolation functions. The specific mathematical expressions of these functions can be found in dedicated textbooks (see [34]). A continuous piecewise linear approximation of the continuous function ψ(x) is defined through Equation (6). The advantage of this approach is that the computational cost depends on the total number of nodes of the mesh and not on the size of the dataset. Discrete expressions of the vector of state variables, background field and covariance are then defined by their values at the grid nodes. Furthermore, the values of the background field at sampling locations can be computed via Equation (6) providing a means to compute the H matrix needed in Equations (2)–(4).

### 2.2. A Metric Suitable for a Sampling On-Demand Strategy ($A_{\eta }$ Criterion)

A metric for the posterior covariance has to be selected. In this work we propose a modifed A criterion that well suits to the sampling on-demand paradigm. A criterion implies the minimization of the trace of the posterior covariance matrix $\hat{\Sigma }$. The optimization process, in this way, aims at decreasing the overall uncertainty over the whole operative area. We propose a new criterion, named $A_{\eta }$, with the cost function to be minimized created on the basis of a map of desired uncertainty provided by the operator. The cost function is defined as

(7) $$J_{\eta }=\sum_{i=1,\;i\in \Delta }^{N}\left({\hat{\sigma }}_{i,i}-\eta_{i}\right),\;\;\;\;\Delta =\left\{k:{\hat{\sigma }}_{k,k}-\eta_{k}>0\right\}$$

where ${\hat{\sigma }}_{i,i}$ is a diagonal element of $\hat{\Sigma }$ and ***η*** is the vector of the desired posterior objective variances of the mesh nodes provided by the user to be achieved by the minimization. The sum is over the number of grid nodes *N* (in the sum we include only the elements in which the posterior variance value ${\hat{\sigma }}_{k,k}$ is larger than the relative desired objective variance $\eta_{k}$). This criterion produces observations reducing the uncertainty according to the map provided by the user (***η***). The desired uncertainty map provides the user with a way to drive the sampling campaign defining both the desired amount of reduction of the uncertainty and the geographical distribution of the resulting $\hat{\Sigma }$ (more information about the field may be required in some areas than in others). These features make the $A_{\eta }$ criterion particularly appealing for the on-field design of missions in which it is mandatory to achieve a certain degree of uncertainty only in some specific areas by means of a limited number of available assets.

## 3. Optimization Procedure for Multiple Gliders

It remains to design an algorithm to minimize Equation (5) with the selected $A_{\eta }$ criterion by planning the glider observations. The created paths must respect glider operational constraints and have to take into account the effects of the ocean flow field on the vehicle navigation. Furthermore, additional geometric constraints need to be included to avoid sensor clustering problems and to produce “smooth” routes.

Solving Equation (5) implies the solution of a nonlinear optimization problem, with the nonlinearity being introduced by the fact that the posterior covariance $\hat{\Sigma }$ is nonlinear with respect to the observation matrix H, which depends on sampling locations, through Equations (3) and (4). These problems are hard to solve when a cost function is too complicated or not differentiable. Soft computing optimization methods offer a possible solution to the problem. Soft computing embeds those techniques that exploit tolerance for precision and uncertainty to provide approximate but timely solutions to computationally hard problems.

To solve our problem, we propose a method based on Simulated Annealing (SA) [30]. Simulated Annealing is a random search method that perturbs the search parameters ***θ*** to minimize a cost index *J* as a function of ***θ***. The perturbations are always accepted if they cause a decrease of *J*. To explore the solution space and avoid local minima, the perturbations are probabilistically accepted if they make *J* increase. This acceptance probability is proportional to the so-called temperature parameter *T* and decreases with time passing (*T* decreases as in the annealing process in metallurgy giving the inspiration to the algorithm). The algorithm [30] converges in the limit to a globally optimal solution with probability 1.

In our problem, by planning different glider paths we define different sets of observations ($\psi^{o}$). At each SA step, new paths are built from which the $\psi^{o}$ are computed. By using Equation (6), the H matrix is computed and then, by using Equations (4) and (3), the index $J_{\eta }$ is computed. The change in the cost function can be evaluated and the next step is run. The process goes on until a pre-defined timeout has expired or until some conditions on the cost are met.

Several parameters/constraints related to the glider navigation features influence the creation of paths:

- number of gliders ($N_{g}$) and their starting locations;
- nominal glider surge speed ($V_{g}$);
- total mission time ($T_{a}$), time between two waypoints ($T_{g}$) (the number of waypoints is therefore $N_{w}=T_{a}/T_{g}$)
- the time between consecutive surfacings ($T_{s}$).

The glider paths are described as a series of waypoints that the gliders try to reach through straight-line segments. In an operative way, to create these paths, SA perturbs the commanded headings (***θ*** parameters of our optimization) of the gliders at waypoints, that is $\chi_{i=0,\ldots ,N_{w}-1}^{j=1,\ldots ,N_{g}}$ (where index *i* runs on the waypoints, i=0 corresponds to the starting location and *j* runs on the gliders). Tentative paths are then built by starting from the glider initial positions. For each heading a straight line of length $L_{g}$ ($L_{g}=T_{g}\times V_{g}$) is planned to define the location of the next waypoint; the procedure is repeated until $N_{w}$ waypoints are produced for each vehicle.

### 3.1. Perturbation Strategy of the Simulated Annealing Parameters

A key point of the Simulated Annealing optimization is the perturbation strategy that modifies the parameters. In our case, we adopt a perturbation law in which the magnitude of the perturbation values depends on the current annealing temperature *T*: as the temperature decreases, the perturbations values are decreased. The underlying idea is that when the temperature is low the geometry of the glider paths has to be modified in a slighter way. The paths, in fact, have likely already reached an almost “optimal” shape, and the small adjustements aim to locally explore the search space to further improve the solution in our constrained scenario (see Section 3.3 for the geometric constraints on the created paths). Experimental results with different datasets demonstrate this strategy can produce better solutions (lower values of the cost function) in the addressed problem than using a perturbation policy in which the magnitudes are independent of *T*.

A new heading $\chi_{i+1}$ is produced as follows

(8) $$\chi_{i+1}=\chi_{i}+\beta \;K_{t}\;\chi_{max}+\;\delta \;K_{t}\;\chi_{min}$$

where $\chi_{i}$ is the heading at the previous step, $\chi_{i+1}$ is the heading after the perturbation, $\chi_{max}$ and $\chi_{min}$ are the maximum and minimum allowed perturbations, *β* and *δ* are two values extracted randomly from standard normal distributions. $K_{t}$ is a value depending on the Simulated Annealing temperature *T* as $K_{t}=\epsilon \log_{10}\left(T/T_{0}\right)+1$ with *ϵ* a constant and $T_{0}$ the starting annealing temperature.

### 3.2. Influence of Water Currents

Water currents highly influence the glider navigation due to the low maximum horizontal speed achievable by the vehicles. The tentative waypoints may not be reached, and, in general, are different from the real ones due to the influence of water flows. For this reason, in the optimization process, we modify the tentative waypoints on the basis of the predictions provided by a mathematical model of the water current field following a procedure detailed in [28]. Water flow model predictions are depth averaged up to the maximum diving depth of the gliders. To compute a realistic trajectory of each *j*th glider between two consecutive waypoints $\left\{x_{i}^{j},x_{i+1}^{j}\right\}$, we consider a point-like model of the platform subjected to a velocity field resulting from adding the local current field $v_{c}\left(x\right)$, produced by the water flow model, to the nominal speed $V_{g}$ in the heading direction $\chi_{i}^{j}$. A heading correction is applied at every time interval determined by the surfacing time parameter $T_{s}$. At each surfacing, the glider *j* estimates the current field by comparing the simulated surfacing position $x_{S}^{j}$ (including the effects of the predicted currents) with that expected from dead-reckoning, ${\hat{x}}_{S}^{j}=x_{S0}^{j}+v_{S0}^{j}T_{s}$, where $x_{S0}^{j}$ and $v_{S0}^{j}=V_{g}\left[\sin\left(\chi_{S0}^{j}\right),\cos\left(\chi_{S0}^{j}\right)\right]$ are respectively the location and velocity derived from previous surfacing point. The current field is then estimated by

(9) $${\hat{v}}_{c}=\frac{x_{S}^{j}-{\hat{x}}_{S}^{j}}{T_{s}}$$

and it is used by the glider to correct the heading to navigate to the tentative waypoint $x_{i+1}^{j}$. The new $\chi_{S0}^{j}$ will be computed as

(10) $$\chi_{S0}^{j}=\tan^{-1}\left(\frac{v_{SW_{x}}^{j}-{\hat{v}}_{c_{x}}}{v_{SW_{y}}^{j}-{\hat{v}}_{c_{y}}}\right)$$

with $v_{\mathrm{SW}}^{j}$ being the speed vector of the glider in the path from the current surfacing point to the next tentative waypoint $x_{i+1}^{j}$, assuming the nominal glider speed $V_{g}$ and is computed as

(11) $$v_{\mathrm{SW}}^{j}=V_{g}\frac{\left(x_{i}-x_{S}\right)}{\left|\right|x_{i}-x_{S}\left|\right|}$$

The surfacing position may differ from the tentative waypoint $x_{i+1}^{j}$ after the navigation time $T_{g}$. In this case, $x_{i+1}^{j}$ is updated with this surfacing location and the process is repeated for the next navigation segment $\left\{x_{i+1}^{j},x_{i+2}^{j}\right\}$. Thus, final waypoints defining the mission are determined from the surfacing locations at each time interval $T_{g}$ defined by the navigation time parameter. The procedure ensures that the final set of waypoints are likely to be achievable in real-world missions.

Glider observations with a certain spacing ($D_{s}$) are then located on the designed glider path. This would correspond to the horizontal sampling resolution of the considered glider.

### 3.3. Geometric Constraints on Created Paths

Geometric constraints on the produced glider paths are added with two objectives: to generate “smooth” trajectories for the gliders and to avoid the problem of sensor clustering (large amount of measurements located in restricted areas).

First of all, to avoid sharp turns in consecutive legs of the path, a limit on the minimum angle between two successive segments is fixed. The minimum angle is set to ±30°. The produced $\chi_{i}^{j}$ headings are therefore modified to respect this condition.

To avoid sensor clustering problems and “knots” in the paths two conditions are also imposed:

1. The distance between the different waypoints are required to be larger than a certain minimum distance $D_{1}$. This condition aims at avoiding clustering of the sensors and minimizes possible collisions between vehicles.
2. A second constraint is set on the waypoints belonging to the same path. The waypoints belonging to the same path (with the exclusion of two consecutive ones) are required to be distant from each other by more than $D_{2}$ (with $D_{2}>D_{1}$). This condition aims at avoiding “knots” arising in the paths of gliders potentially hampering an effective search of the solution space during the optimization.

The described constraints are introduced as penalty factors into the cost function. Mathematically, the two constraints are modeled as

(12) $$c_{1}=\left\{\begin{array}{cc} \max_{ij}\left(D_{1}/d_{ij}+1\right) & \;\;if\;\;\max_{ij}\left(D_{1}/d_{ij}+1\right)>2\\ 2 & \;\;if\;\;\max_{ij}\left(D_{1}/d_{ij}+1\right)\leq 2 \end{array}\right.$$

where $d_{ij}$ is the distance between all the produced waypoints and the maximum is considered over all the possible waypoints combinations (i,j) with i≠j. And the second constraint is,

(13) $$c_{2}=\left\{\begin{array}{cc} \max_{ij}\left(D_{2}/d_{ij}+1\right) & \;\;if\;\;\max_{ij}\left(D_{2}/d_{ij}+1\right)>2\\ 2 & \;\;if\;\;\max_{ij}\left(D_{2}/d_{ij}+1\right)\leq 2 \end{array}\right.$$

where $d_{ij}$ is the distance between the waypoints belonging to the path of a single glider and the maximum is considered over all the possible combinations of non-consecutive waypoints (i,j) belonging to the vehicle paths. We set a minimum value of 2 for $c_{1}$ and $c_{2}$ when $d_{ij}$ are larger than the required distances to avoid that the SA forces an increase of $d_{ij}$ when all $d_{ij}$ are larger than the set distances. In that case, in fact, the penalty function does not change when $d_{ij}$ is larger than the requested constraints, thus not modifying the objective cost function.


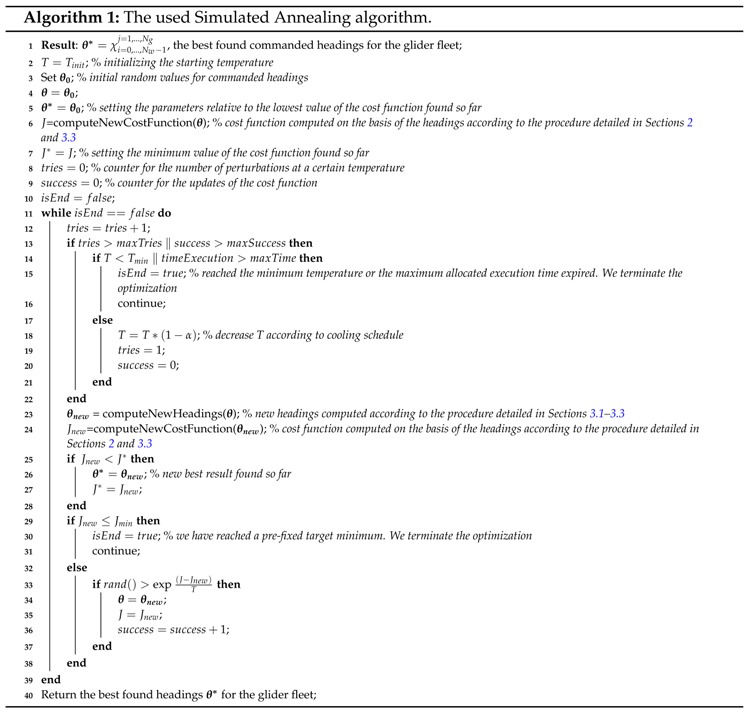


The penalty functions are added to the cost $J_{\eta }$ to be minimized. Thus, the final cost function *J* comprises two components:

(14) $$J=J_{\eta }+\gamma_{J}J_{c}$$

with $J_{\eta }$ defined in Equation (7) and representing the part related to the minimization of the posterior covariance, $J_{c}$ is the cost due to the geometric constraints and $\gamma_{J}$ a normalization factor. $J_{c}$ is taken as the maximum value of the two geometric penalty components, that is $J_{c}=\max\left(c_{1},c_{2}\right)$. In this way, the Simulated Annealing takes into account the geometric constraints directly by optimizing the resulting *J* cost index.

The resultant Simulated Annealing algorithm is reported in Algorithm 1.

## 4. SoDDS: A Matlab Toolbox for Sampling on Demand and Decision Support

The optimization algorithm described in the previous sections has been implemented in Matlab (^®^MathWorks). To ease its daily use in the CMRE Command and Control Room during monitoring campaigns it has been integrated in the SoDDS (Sampling on-Demand and Decision Support) toolbox. SoDDS is provided with the capability of automatic data download and, through graphical user interfaces, supports the operators in planning missions. Effective tools to support the operators in planning and conducting oceanographic missions are more and more needed due to the increasing complexity of requested tasks (see [35]). In the next sections we will describe how SoDDS is organized and its features.

### 4.1. Automatic Download of Ocean Field Forecasts from MyOcean

#### 4.1.1. The MyOcean Service

The MyOcean service [32] is a marine core service that aims at providing users the best generic information available on the state of the oceans. MyOcean (2009–2012) and now MyOcean2 (2012–2014) are committed to develop and run an European service based on a worldwide capacity for ocean monitoring and forecasting, using observation data, modeling and assimilation systems. The service is particularly valuable since it provides a single catalog for all the products, like analyses, reanalyses, and forecasts. MyOcean is a gateway to products provided by external research centres, where each product is a collection of different datasets. Each dataset contains different variables (sea currents, temperature, salinity, *etc.*). Within a product and a dataset, the user can download a portion of the data using the motu-client, as described in next subsection.

#### 4.1.2. Motu-Client: The Python Script for Downloading Forecasts

Motu-client is a Python script (mou-client.py) that runs from the shell and that allows to download the sub-dataset of interest within a specific dataset contained in a particular product. Each sub-dataset is characterized by: the spatial range (expressed as a rectangular area), the temporal range and the depth range. Doing this, the user is able to download only the data he is interested in, thus saving storage disk space and gaining in downloading speed. The parameters that can be specified from the command line of a motu-client call are shown in Table 1. The sub-dataset is retrieved as a NetCdf binary file. Next section describes the implemented Matlab toolbox, named Sampling on-Demand and Decision Support.

### 4.2. The SoDDS Matlab Toolbox Structure

SoDDS wraps the Python motu-client script and provides the operator two graphical user interfaces to simplify the mission planning task. SoDDS supports the operators throughout the planning and analysis of the mission. The mission planning is made up of four steps.

#### 4.2.1. Step 1—Data Download

The data downloader is a Matlab wrapper to Python motu-client script. Therefore this function has to collect all the required motu-client parameters and then perform a call to it. Key advantages of the Matlab wrapper over motu-client are twofold: it comes with a graphical user interface for defining the region of interest of the sub-dataset, and previously defined regions can be easily re-used in subsequent calls (see next step).

#### 4.2.2. Step 2—Setting the Region of Interest GUI (roiGUI)

**Figure 1:**
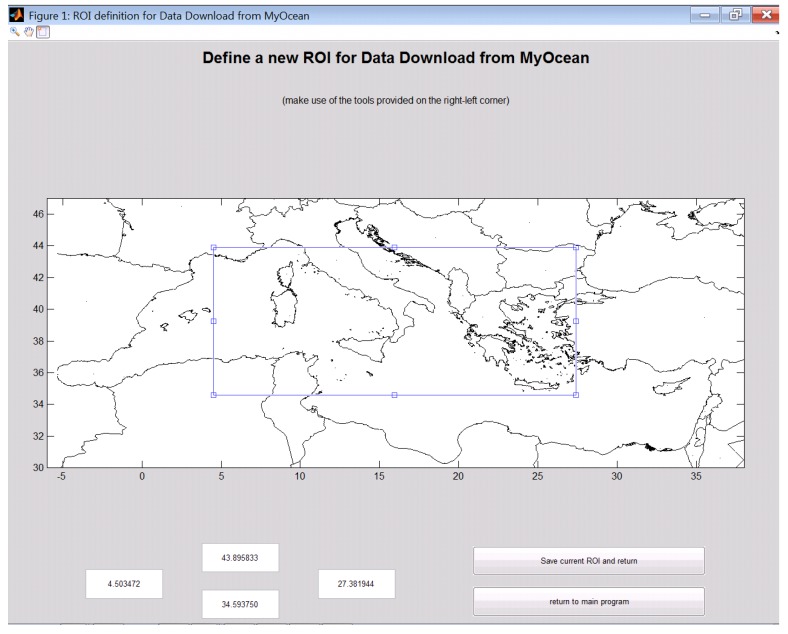
The Region Of Interest (ROI) definition GUI.

As stated above, motu-client requires the user to specify spatial portion of the dataset he wants to download and thus it requires the minimum and maximum longitude and the minimum and maximum latitude of the Region Of Interest (ROI). This means that the user must select a rectangular bounding box of the sampling area of his interest. The GUI, shown in Figure 1, allows the user to zoom and pan a picture showing the coastline of the Mediterranean Sea and to drag the ROI (the blue rectangle in that figure). The ROI can also be resized by using the mouse or by entering the values in the four edit boxes provided in the bottom part of the GUI (useful when the right bounding box is exactly known beforehand). When the user is satisfied with the selected ROI he can save it on disk for future use (the system asks a unique name for it before saving). Each ROI is defined by the attributes provided in Table 1.

**Table 1:** ROI Descriptor.

Attribute	Meaning
area	Area, as defined in MyOcean (“Med”, “ArticOcean”, “NorthSea”, *etc.*)
lonMin	Minimum Longitude
lonMax	Maximum Longitude
latMin	Minimum Latitude
latMax	Maximum Latitude
vars	lField variable to download (it can be “C” for currents, “T” for temperature, and “S” for salinity, or a combination like “CT” or “CS”)

#### 4.2.3. Step 3—The Covariance Matrix Generation

Once the sea currents and the field to be optimally sampled have been downloaded from MyOcean, the covariance matrix has to be computed. For instance, when considering the Sea Surface Temperature (SST), the 4D dataset (3 spatial dimensions plus time) must be masked to exclude land regions. Then it has to be averaged along the z direction (the depth). At this point the prior covariance matrix can be easily computed, following the procedure described in Section 5.

#### 4.2.4. Step 4—The Mission Constraints GUI (mcGUI)

**Figure 2:**
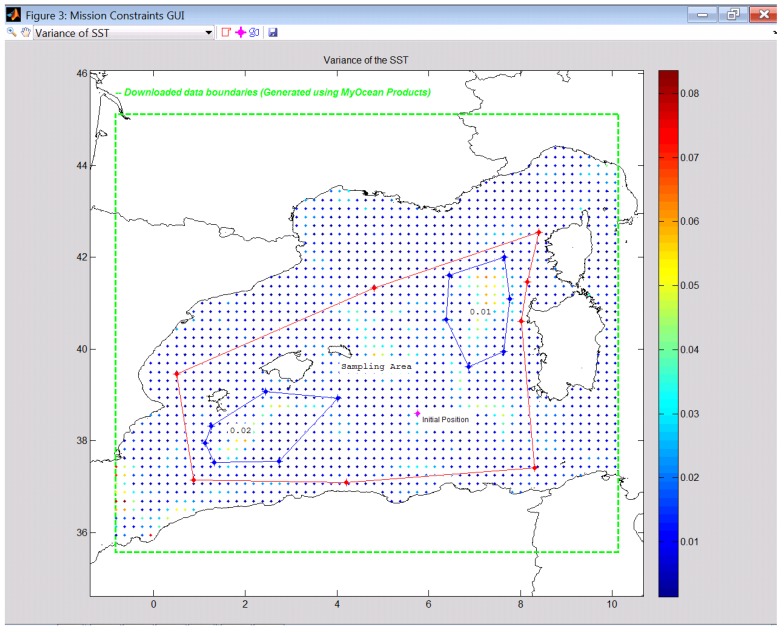
The mission constraints GUI (Generated using MyOcean products). The variable displayed is the variance of the Sea Surface Temperature (the colormap on the right shows its range of variability). In the sampling area delimited by a red polygon, the initial deployment position of one glider is indicated by a magenta spot. The two regions delimited by blue polygons area areas in which a target variance has been defined by the user (in the first, on the left, the target value is 0.02, while in the second, on the right, the target is 0.01).

The mission constraints GUI (Figure 2) is an interface that shows the downloaded ocean field (Temperature, Salinity, *etc.*) and its variance and standard deviation, for the ROI specified in previous step. Moreover it allows the user to easily (*i.e.*, graphically) specify the following information:

- the sampling area;
- the gliders’ deployment positions;
- the target variance.

For example, the red polygonal line shown in the mission constraints of Figure 2 represents the sampling area (it has been drawn by hand by using the mouse with the user being able to drag each single vertex to better define the area). It also displays magenta spots representing the glider deployment positions. Finally it shows two blue polygonal regions, reporting a number. These numbers represent the target posterior variance, *i.e.*, the target value to be achieved by optimal planning of the glider paths. Next section will show some results obtained by running the SoDDS toolbox.

## 5. Experimental Results

**Figure 3:**
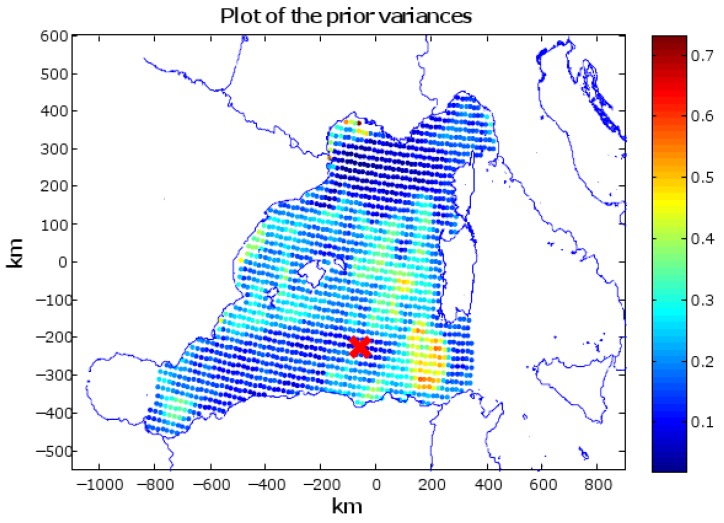
Values of the prior variances of the grid mesh nodes (diagonal values of the prior covariance matrix **Σ** in the area of study). The red marker represents the starting position used for the gliders (38.0654° N, 5.16° E).

To investigate the performance and the features of the proposed approach we applied our method to study the temperature field at 50-m depth in an area of the Western Mediterranean Sea, specifically covering almost all the waters west of the coasts of Sardinia and Corsica (see Figure 3). Background statistics have been built on the basis of a time series of monthly reanalysis of the temperature fields at 50 m depth resulted from the Mediterranean Forecasting System (MFS), a component of MyOcean.

On the basis of the ergodic assumption (see Section 2), we computed the prior covariance matrix **Σ** and the known background $\bar{\psi }\left(x\right)$ used in our tests. Since we apply our algorithm to plan a mission in January 2012, the known background is computed by averaging the values of 12 historical series of average temperature data provided by the model for the months of January belonging to 12 consecutive years (2000-2011). **Σ** is obtained as the sample covariance and was then modified through a transformation named *shrinkage* [36]. This operation tends to pull the most extreme coefficients towards more central values, thus systematically reducing estimation error where it matters most. A model of the water currents is also used to produce an estimate of the current field averaged in the first 200 m (maximum depth reached by the gliders) and represents a prediction of the current field the vehicles encounter during their mission.

A grid was computed covering the area of study with a spacing in latitude of 0.083°and in longitude of 0.1875°. A Cartesian reference frame is also added with its origin fixed to the center of the area of study. In our tests, we consider the gliders are all deployed at the same location (38.0654° N, 5.16° E). This condition represents a common situation in operational scenarios in which the vessel starts deploying the vehicles at a preset deployment site. It also represents the most complex situation from the point of view of respecting the required geometric constraints on paths since the gliders move (at least at the beginning of the mission) in nearby areas. Prior values of the grid mesh in the operation area are shown in Figure 3 along with the gliders’ deployment location.

The parameters used to characterize the glider mission are reported in Table 2 and are typical of real glider missions conducted by NATO STO Centre for Maritime Research and Experimentation (CMRE) in operations at sea.

**Table 2:** Parameters for the missions of gliders.

$V_{g}$	0.35 (m/s)	nominal gliders surge speed
$T_{a}$	12 (days)	total mission time
$T_{g}$	48 (h)	time between two waypoints.
		The number of waypoints $N_{w}$ is therefore equal to 6
$T_{s}$	6 (h)	time between surfacings
Currentfield	μ=0.1 (m/s) σ=0.07 (m/s)	mean and standard deviation value of the current field

It is important to remark that the current field is in some areas comparable with the glider surge speed and thus strictly constrains the vehicle mobility. This is taken into consideration by the optimization algorithm as previously described.

Concerning the algorithm, important parameters to be set are the values of the geometric constraints (Section 3.3) strongly dependent on the geometry of the addressed scenario. We start defining $l_{g}=V_{g}T_{g}$ (theoretical distance covered between two waypoints), in our case $l_{g}=60.480$ km. On the basis of this value, we set $D_{1}=l_{g}/3$ and $D_{2}=l_{g}$. Furthermore, the observation error covariance matrix is assumed diagonal of the form vI, with I identity matrix and the value *v* equal to the average of the diagonal values of the prior covariance matrix, **Σ**.

The used Simulated Annealing algorithm is described in Algorithm 1 and was implemented in Matlab (${}^{©}$MathWorks) and was run on an Intel Core2^®^ Quad CPU Q6600 @2.4 GHz with 4 GB of RAM memory. The algorithm code was adapted from [37]. The optimization terminates and provides the so far best solution when one of the following conditions occurs: $J_{\eta }=0$, that is the objective of the optimization is completely reached, after a certain amount of execution time or if the temperature *T* becomes lower that a certain $T_{min}$. The used parameters in the algorithm are reported in Table 3.

**Table 3:** Parameters used for the optimization algorithm.

$T_{init}$	5	starting temperature
$T_{min}$	$10^{-8}$	minimum temperature
*α*	0.15%	temperature decrease as decrement in percentage of the current temperature value
maxTries	50	maximum number of parameter perturbations at a certain temperature before its decrement
maxSuccess	20	maximum number of updates of ***θ*** at a certain temperature triggering a temperature decrement
$J_{min}$	2	minimum value of the cost function which, if reached, caused the termination of the algorithm. The value 2 means $J_{\eta }=0$ and the geometric constraints satisfied
$\chi_{max}$	1.2217 (rad)	maximum value of the perturbations of the glider heading (70 °)
$\chi_{min}$	−1.2217 (rad)	minimum value of the perturbations of the glider heading (−70 °)
*ϵ*	0.2	parameter used in the perturbation strategy
$\beta_{J}$	1	weighting factor for *J* index

**Figure 4:**
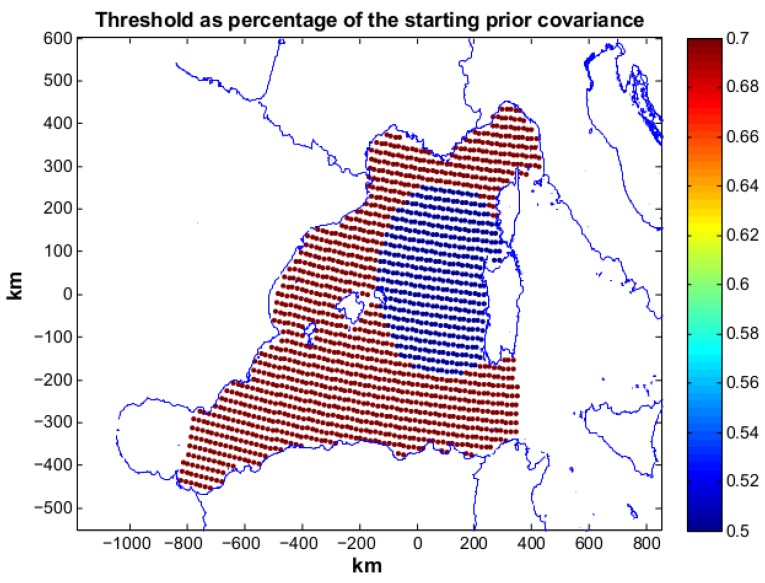
Threshold as percentage of the starting prior variances for scenario 2. The desired posterior is 70% of the prior in all of the area of study except that in a circular area with a radius of 230 km in front of the Sardinia coast where the desired posterior is requested to be 50% of the relative prior.

Three different scenarios are here presented and discussed. Each scenario is characterized by a different objective threshold η(x) set by the user reflecting different mission requirements. In *scenario 1* the threshold values are set to 70% of the relative prior variance values in the entire area of study, that is $\{\eta_{i}=0.7\sigma_{ii}$, for $i=1\ldots N_{c}\}$, with $N_{c}$ being the number of cells in the grid mesh. In *scenario 2* threshold values are equal to 70% of the relative prior variance values throughout the operation area except that in a circular area with a 230 km radius centered west of Sardinia where the threshold values are equal to 50% of the relative priors (see Figure 4). In *scenario 3* threshold values are equal to 50% of the relative prior variance values all over the operation area.

**Figure 5:**
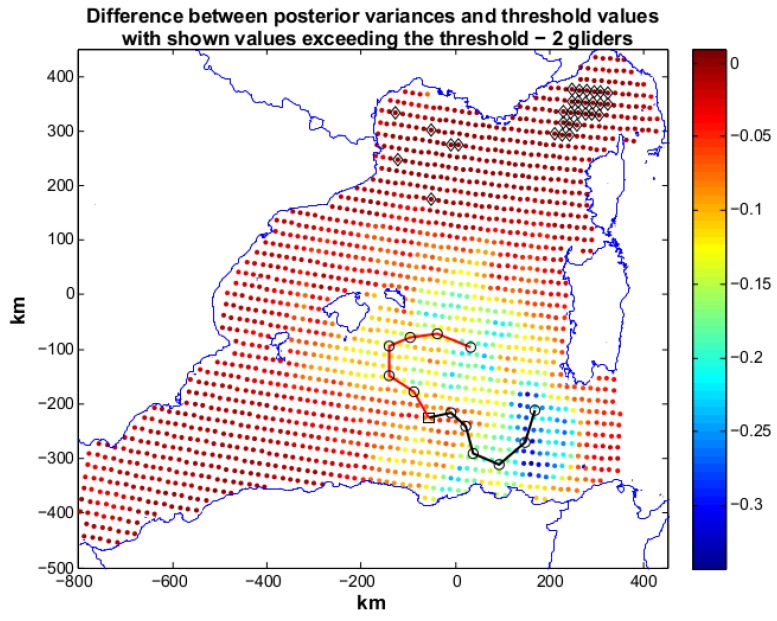
Scenario 1: planned paths for two gliders. The solution is characterized by $J_{\eta }=0.0828$ and $J_{c}=2$. In the figure we show also the difference between posterior variances and relative values of the objective threshold. Black markers toward the north-east part of the operation area indicate locations whose posterior was not lowered enough. These locations are limited in number proving the optimization produced a solution close to the optimum.

**Figure 6:**
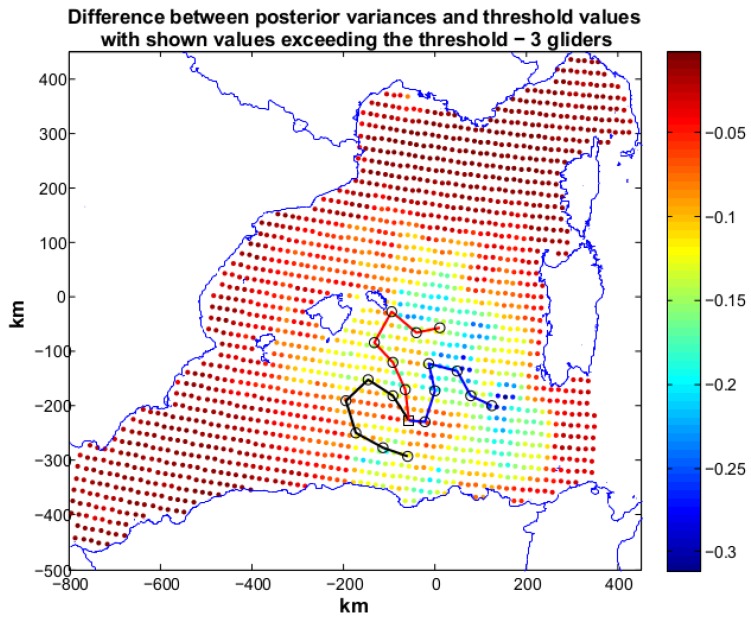
Scenario 1: planned paths for three gliders. The solution is characterized by $J_{\eta }=0$ and $J_{c}=2$. In the figure we show also the difference between posterior variances and relative values of the objective threshold. All the posterior variances in the operation area respect the requirements of the mission.

Scenario 1 represents a situation in which the user sets a desired posterior variance which is moderately lower than the prior. A first annealing with two gliders, $R_{2}$, is run and terminates with the following indices: J=2.0828, with $J_{\eta }=0.0828$ and $J_{c}=2$. The latter value means that the geometric constraints are fully respected. From our experience we have seen that the geometric constraints are in general easily respected if the number of deployed gliders is limited (<4). The resulting paths are shown in Figure 5 along with the difference between posterior variances and the target threshold. The black markers show grid nodes (30 in total) characterized by posterior variances that are higher than the relative $\eta_{i}$. In particular, these locations are present in the north-east part of the area of study. $J_{\eta }$, however, has a low value, showing the minimization target is close to be reached. An annealing with three gliders, $R_{3}$, was then started and the index reaches the target value, that is $J_{\eta }=0$ and $J_{c}=2$. The produced paths are reported in Figure 6. The three paths cover (without mutual intersections) the region in the center of the area of study and the planned measurements succeed in lowering the posterior up to the required values. The use of three gliders meets the target set by the user. However, the cost value produced by $R_{2}$ is close to the objective and suggests that two gliders are also a viable possibility to achieve the mission requirements. To plan an at sea mission, a cost-benefit analysis would drive the decision on deploying two or three vehicles.

**Figure 7:**
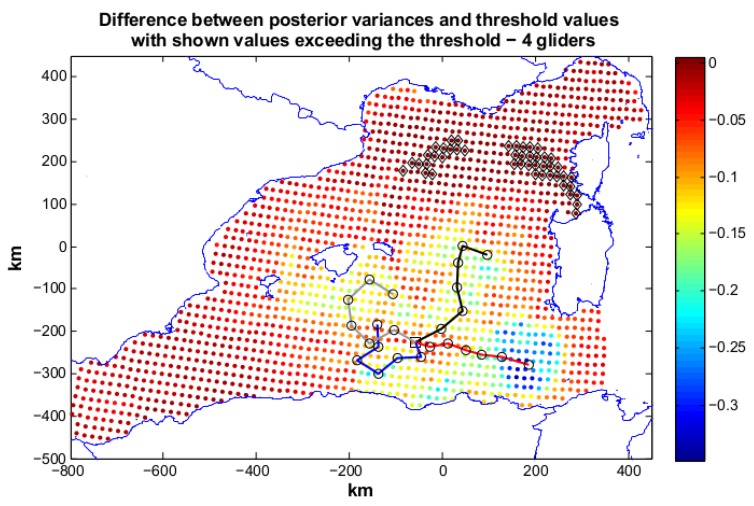
Scenario 2: planned paths for four gliders. The solution is characterized by $J_{\eta }=0.22$ and $J_{c}=2.24$. In the figure we also show the difference between posterior variances and relative values of the objective threshold. The solution almost reaches the mission target. More gliders are needed to reach the mission objective.

**Figure 8:**
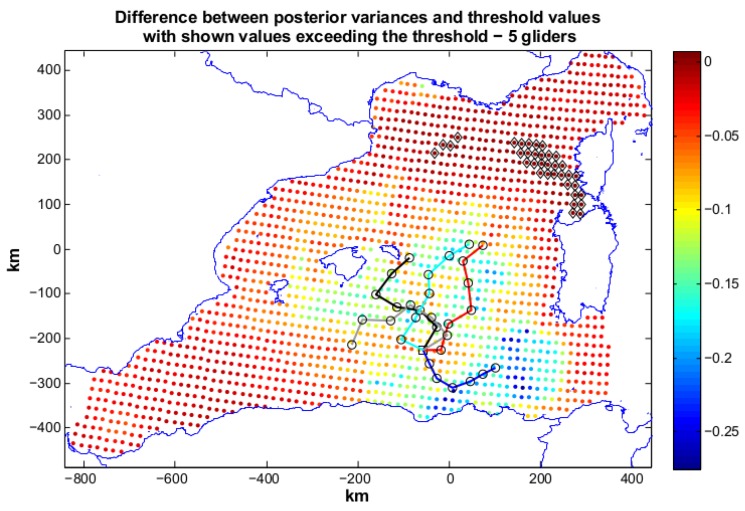
Scenario 2: planned paths for five gliders. The solution is characterized by $J_{\eta }=0.09$ ($J_{c}=2.06$). In the figure we also show the difference between posterior variances and relative values of the objective threshold. Black markers toward the north-east part of the operative area indicate locations whose posterior is still above the required threshold. The use of five gliders is considered satisfying, since we are quite close to the achievement of our goal (no black marks).

In scenario 2 the requirements are more demanding, since for the area located around Sardinia a lower posterior covariance is requested(50% of the prior) (see Figure 4). This may be a frequent case in an operative scenario, where more information content is needed to be acquired in specific areas of interest. Our sampling on-demand strategy addresses this need explicitly. An optimization with four gliders, $R_{4}$, was launched. $R_{4}$ was not able to reach the mission objective and produces a solution with $J_{\eta }=0.22$ ($J_{c}=2.24$). The produced paths are reported in Figure 7. Some locations above the set threshold are present in the area close to Sardinia (50% area) and far from the glider trajectories. $N_{g}=5$ was then explored, and the annealing $R_{5}$ was run with resulting $J_{\eta }=0.09$ and $J_{c}=2.06$. $R_{5}$ finds a good solution to our problem almost completely respecting the geometric constraints. The paths are shown in Figure 8: three gliders head toward the coast of Sardinia to lower the posterior of the 50% area. Some locations in the north-east zone still present a posterior not satisfying the mission objectives due both to the required strong posterior covariance decrement in the area around Sardinia and to their distance from the locations reachable by the glider paths. However, these grid nodes present posteriors close to the sought values as the small value of $J_{\eta }$ shows. The solution using 5 gliders can be therefore considered good and would have been chosen for planning an at sea mission.

**Figure 9:**
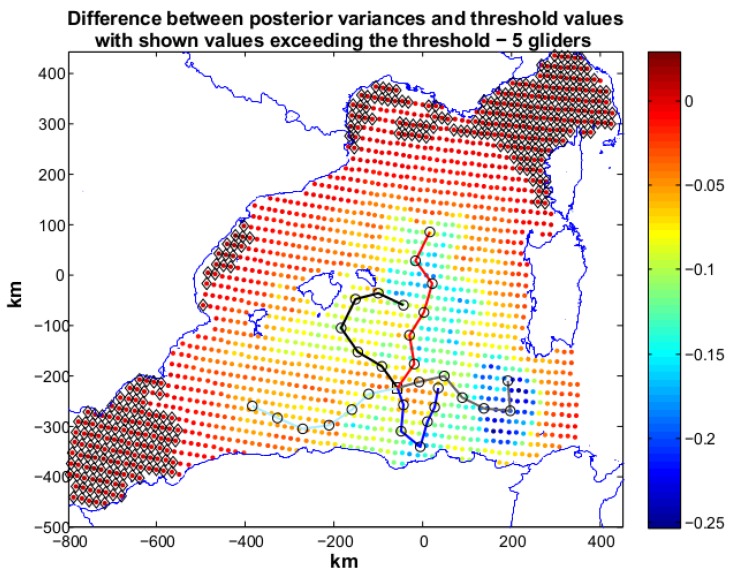
Scenario 3: planned paths for five gliders. The solution is characterized by $J_{\eta }=1.67$ ($J_{c}=2$). In the figure we show also the difference between posterior variances and relative values of the objective threshold. The high number of black markers toward the north-east and south-west parts of the operative area indicates posteriors for which gliders did not succeed in reducing enough failing to meet the target variance. More samplings (gliders) are therefore needed.

**Figure 10:**
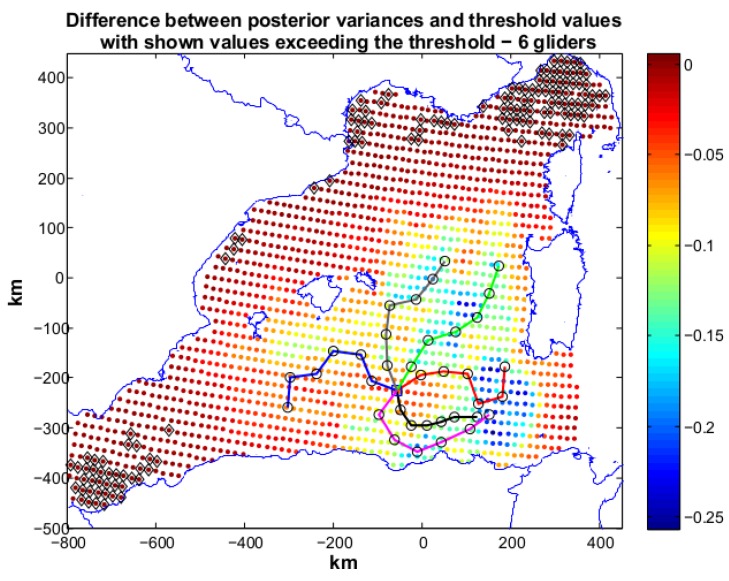
Scenario 3: planned paths for six gliders. The solution is characterized by $J_{\eta }=0.14$ and $J_{c}=2$. In the figure we show also the difference between posterior variances and relative values of the objective threshold. Black markers indicate posteriors that gliders did not succeed in lowering enough. These locations are limited in number showing the optimization has found an acceptable solution to the problem.

Scenario 3 is the most demanding one in terms of objective threshold. The desired posterior variances is set as 50% of the prior variances over all the operative area. Given the demanding objective, we used five gliders in the optimization ($R_{5}$) and the algorithm terminates with $J_{\eta }=1.67$ ($J_{c}=2$). The paths produced by $R_{5}$ are shown in Figure 9. Locations not reaching the target values are present in the north-east and south-west parts of the operative area. A new optimization with 6 gliders was then run. The solution (see Figure 10) of this optimization is characterized by $J_{\eta }=0.14$ and $J_{c}=2$.

6 gliders succeeded in lowering the posterior even if some areas in the north-east and south-west corners could not be influenced as we required. This is due to the distance of these areas from the glider paths and to the requested low value of posterior. In this scenario, the solution with six gliders results as the only reasonable candidate to provide a good solution to our problem.

The computational time needed by our approach depends on the size of the region and the number of vehicles. In our experiments it was of the order of hours (for six gliders *maxTime* was considered 5 hours), an acceptable time for our needs . For grids with a larger number of points or an increased number of gliders, an optimization of the code and its porting to a supercomputer to parallelize its execution would speed up it significantly.

## 6. Conclusions

This paper describes an optimal mission planning algorithm for a fleet of gliders based on the sampling on-demand paradigm. In the sampling on-demand strategy, the user sets quantitatively the requirements related to the uncertainty over an area of interest that needs to be achieved by means of measurements taken by observing assets. In general, the set uncertainty can vary from one region to another of the area of study according to the scientific/operational requirements of the mission. This may be due, for instance, to the fact that some areas are considered more important to explore than others. This paradigm addresses appropriately the requirements of real operative scenarios.

A new optimality criterion suited to the sampling on-demand paradigm, called $A_{\eta }$, has been introduced. The sampling metric related to the $A_{\eta }$ criterion is mimimized by planning the sampling locations (paths) of the gliders. This mimization produces optimal paths for the vehicles (a series of waypoints). To solve this complex nonlinear optimization problem, we proposed and discussed an algorithm based on Simulated Annealing. The algorithm takes into account constraints due to the desired geometry of the paths and also the constraints introduced by vehicle dynamics and by the influence of the ocean currents on vehicle navigation.

To pursue the idea of creating an operational tool for scientists, we have integrated the proposed sampling on-demand algorithm in a Matlab toolbox, named Sampling on Demand and Decision Support (SoDDS). SoDDS is capable of downloading the forecasts of the ocean fields of interest and the ocean currents from the public available MyOcean repository. MyOcean service allows to standardize the access to ocean field forecasts generated by different and heterogeneous research organizations. The tool provides graphical user interfaces allowing a user-friendly definition of the sampling area and the definition of the desired target uncertainties. This tool provides an integrated, effective and operational system for glider operators to ease the whole mission planning process and post-mission analysis.

Results of using SoDDS tool in the considered scenarios prove that our methodology is effective in planning “smooth” glider paths minimizing the $A_{\eta }$ cost function, at the same time satisfying the mission constraints.

In addition, a preliminary version of this planner has been applied to one glider showing a gain of the collected information for assimilation purposes [16].

Our approach can therefore support marine scientists to plan effectively sampling missions at sea characterized by target posterior uncertainty different in quantity in different geographic areas. SoDDS can also be used in combination with other decision support tools, such as the glider decision support tool described in [38], or with a fuzzy rule-based system [39], within a glider command and control room.

In the sampling on-demand paradigm, however, one important question to be solved is the minimum sufficient number of gliders to achieve the requirements of the mission. With this information, scientists could achieve their operational objectives with the minimum number of vehicles and reduce the number of deployed assets (costs and complexity reduction). Since the amount of time an optimization potentially requires may limit the scope of applicability, future work will address how to find this minimum number without a time-expensive, “brute” force approach, in which the optimization is run with all the possible numbers of gliders until the mission objectives are met.

## Abbreviations

The following abbreviations are used in this manuscript:

DOAJ: Directory of open access journals

TLA: Three letter acronym

LD: linear dichroism
